# Prevalence of Diabetic Foot Disease in Patients with Diabetes Mellitus under Renal Replacement Therapy in Lleida, Spain

**DOI:** 10.1155/2016/7217586

**Published:** 2016-04-14

**Authors:** Montserrat Dòria, Verónica Rosado, Linda Roxana Pacheco, Marta Hernández, Àngels Betriu, Joan Valls, Josep Franch-Nadal, Elvira Fernández, Dídac Mauricio

**Affiliations:** ^1^Department of Endocrinology & Nutrition, University Hospital Arnau de Vilanova and Santa Maria, Alcalde Rovira Roure 80, 25198 Lleida, Spain; ^2^Department of Nephrology, University Hospital Arnau de Vilanova and Santa Maria, Alcalde Rovira Roure 80, 25198 Lleida, Spain; ^3^Biostatistics and Epidemiology Unit, Biomedical Research Institute of Lleida, Alcalde Rovira Roure 80, 25198 Lleida, Spain; ^4^Department of Mathematics, Autonomous University of Barcelona, 08193 Bellaterra, Spain; ^5^DAP-Cat Group, Unitat de Suport a la Recerca Barcelona Ciutat, Institut Universitari d'Investigació en Atenció Primària Jordi Gol (IDIAP Jordi Gol), Sardenya 375, 08006 Barcelona, Spain; ^6^CIBER of Diabetes and Associated Metabolic Diseases (CIBERDEM), Instituto de Salud Carlos III (ISCIII), Monforte de Lemos 3-5, 28029 Madrid, Spain; ^7^Primary Health Care Center Raval Sud, Gerència d'Àmbit d'Atenció Primària Barcelona Ciutat, Institut Català de la Salut, Avinguda Drassanes 17-21, 08001 Barcelona, Spain; ^8^Department of Endocrinology & Nutrition, Health Sciences Research Institute and Autonomous University of Barcelona, University Hospital Germans Trias i Pujol, Carretera Canyet, S/N, 08916 Badalona, Spain

## Abstract

*Aim*. To assess the prevalence of diabetic foot and other associated conditions in patients with diabetes mellitus under renal replacement in the region of Lleida, Spain.* Methods*. This was an observational, cross-sectional study of 92 dialysis-treated diabetic patients. Besides a podiatric examination, we explored the presence of cardiovascular risk factors, late diabetes complications, including peripheral neuropathy, atherosclerotic disease, and peripheral artery disease. We assessed risk factors for foot ulceration and amputation by logistic regression.* Results*. Prevalent diabetic foot was found in 17.4% of patients, foot deformities were found in 54.3%, previous ulcer was found in 19.6%, and amputations were found in 16.3%; and 87% of them had some risk of suffering diabetic foot in the future. We observed a high prevalence of patients with peripheral neuropathy and peripheral artery disease (89.1% and 64.2%, resp.). Multivariable analysis identified diabetic retinopathy and advanced atherosclerotic disease (stenosing carotid plaques) as independent risk factors for foot ulceration (*p* = 0.004 and *p* = 0.023, resp.) and diabetic retinopathy also as an independent risk factor for lower-limb amputations (*p* = 0.013). Moreover, there was a temporal association between the initiation of dialysis and the incidence of amputations.* Conclusion*. Diabetic patients receiving dialysis therapy are at high risk of foot complications and should receive appropriate and intensive foot care.

## 1. Introduction

Diabetic foot (DF) is a chronic complication of diabetes mellitus (DM) associated with a high economic and social burden. The lifetime risk of developing a foot ulcer in a patient with diabetes ranges from 15% to 25% [1, 2]. The development of a DF ulcer depends on a constellation of several factors occurring together, and several causal pathways can lead to ulceration [3]. One of these pathways is the triad neuropathy-foot deformity-minor trauma [4]. There are important factors that determine the ulcer outcome, such as peripheral arterial disease (PAD), infection, and patient-related factors, including advanced age and the presence of comorbidities that can affect wound healing or renal replacement therapy [3, 5].

Diabetic nephropathy has been identified as an important risk factor for foot ulceration and amputation [6, 7]. Additionally, dialysis treatment has been reported as an independent risk factor in diabetic patients with chronic kidney disease [8]. About 20% of diabetic patients develop foot ulcers during the first year after initiation of dialysis [9], and the amputation rate is 4% for every year in dialysis therapy [10]. Moreover, renal failure has been reported to independently predict the risk of nonhealing ischaemic and neuroischaemic foot lesions and major amputations [11, 12]. Uraemia has a negative effect on ulcer healing, with nonuraemic patients having a 2.45 increased probability of primary healing of the ulcer [12]. Additionally, end-stage renal disease (ESRD) has a stronger negative effect in diabetic patients with PAD than in those without this complication [11].

The aim of the study was to assess the prevalence of DF and other associated conditions in patients with DM under renal replacement in the region of Lleida, Spain.

## 2. Methods

### 2.1. Design

This was an observational, cross-sectional study conducted between November 2010 and March 2011. All diabetic patients with chronic kidney disease who were receiving replacement therapy (haemodialysis and peritoneal dialysis) from the two existing dialysis centres in the health area of Lleida, a region of Spain with a population around 450,000, were included.

### 2.2. Studied Variables

Study participants were recruited from the only two haemodialysis centres in the area of Lleida, Spain, and those under peritoneal dialysis during their routine clinical visits at the Nephrology Unit of the Hospital Arnau de Vilanova. Patients were considered to have DM if they had ever been diagnosed with the disease or, alternatively, if they were receiving any antidiabetic agent. The type of DM was assigned based on their medical records or clinical data when the diagnosis was missing.

For each participant, data were collected on the following variables: age; gender; type and duration of DM; type and duration of dialysis (i.e., haemodialysis or peritoneal dialysis); presence of cardiovascular risk factors: hypertension, that is, if they had ever been diagnosed with the disease or were receiving any antihypertensive medication; smoking status and dyslipidaemia; and long-term diabetes complications: the presence of any degree of diabetic retinopathy [DR], amaurosis, neuropathy, cerebrovascular disease, and ischaemic heart disease. Moreover, during anamnesis, patients were asked about their previous history of foot ulcers, defined according to the current consensus of the International Working Group on the Diabetic Foot (IWGDF) [13].

All patients underwent a detailed foot examination to identify previous lower-limb amputations (minor or major) and foot deformities. Foot deformity was defined according to the IWGDF recommendations [14], namely, as structural abnormalities of the foot such as hammer toes, mallet toes, claw-toes, hallux valgus, prominent metatarsal heads, residuals of neuroosteoarthropathy, amputations, or other foot surgeries. Peripheral neuropathy (PN) was explored with an ultrabiothesiometer (Me. Te. Da. Srl., IT) applied on the dorsal part of the first toe and considered abnormal when the patient needed >25 V intensity to perceive the vibration. PAD was explored through the ankle-brachial index (ABI), assessed with an ES100X MiniDop® Surgical Doppler (Koven Technology, US). ABI values were classified as normal (from 0.91 to 1.30), moderate ischaemia (from 0.41 to 0.90), severe ischaemia (from 0 to 0.40), and noncompressible because of the presence of calcification (>1.30). Moreover, in patients with an ABI value >1.30, we also explored the pedal or posterior tibial pulse: if any of the pulses was nonpalpable, the patient was classified as having PAD. Finally, a carotid ultrasound was performed using a GE Vivid and BT09 Doppler (General Electric, US) in order to detect subclinical atherosclerosis through morphological examination (i.e., the presence or absence of plaques in any of the territories of the carotid artery) and to calculate the intima-media thickness (IMT) of the common carotid artery. A detailed description of the procedure has been recently published [15]. Briefly, B-mode images of the left and right segments of the common carotid artery were recorded and electronically stored; IMT measurements were performed offline using semiautomatic software with the last (previous to the bulb) centimetres of the common carotid artery, and the mean IMT was obtained by averaging the right- and left-side values.

Based on the ABI and on the reference values for carotid IMT in a Spanish community cohort [16], atherosclerotic disease was classified as follows: AD0, no atherosclerotic disease: ABI > 0.9 and <1.3 and IMT < 90% of the reference interval (RI) for sex and age; AD1, ABI 0.7–0.9 or = 1.3 and/or IMT > 90% of the RI for sex and age; AD2, nonstenotic plaques (<50% lumen stenosis); AD3, ABI < 0.7 or >1.3 and/or stenotic plaques with >50% lumen stenosis.

Patients were classified into four risk groups based on the presence of risk factors according to the IWGDF consensus [13]: (a) group 0: patients without distal sensory neuropathy; (b) group 1: patients with distal sensory neuropathy; (c) group 2: neuropathic patients with some grade of PAD and/or foot deformity; and (d) group 3: neuropathic patients with a history of prior foot ulcer or lower-limb amputation.

The local Ethics Committee from the Hospital Arnau de Vilanova (Lleida, Spain) approved the protocol, and all patients signed a written informed consent form prior to participation. The study was conducted in accordance with the Declaration of Helsinki (1964).

### 2.3. Statistical Analysis

Baseline variables were described by range, mean and standard deviation (±SD) for quantitative variables, or frequency and percentage for qualitative variables. A bivariate analysis was conducted to assess the risk of ulceration and amputation through contingency tables; the significance was assessed with Pearson's Chi-square test and the estimated relative risk and corresponding 95% confidence intervals (CIs) were calculated using exact methods (mid-*p*) and normal approximation (Wald). For quantitative variables, we estimated the mean differences and assessed the significance by performing Student's *t*-test (for normally distributed variables) or Mann-Whitney test (for non-normally distributed variables). In addition, a multivariate analysis was conducted to evaluate the association of the variables with amputation and prognosis value. For this purpose, we used simple logistic regression models adjusted for all the variables that were observed to be individually statistically significantly associated with ulcers or amputations in the bivariate analysis at a *p* value < 0.1. Subsequently, we applied a backwards-stepwise regression algorithm to select the most relevant factors, using Akaike's Information Criterion.

Finally, we studied whether there was a temporal association between the onset of dialysis and amputations. For this, we calculated the absolute and relative frequency (%) for the punctual and cumulative incidence (from the time of first amputation and from the onset of dialysis). Negative values indicate patients with amputations prior to the start of dialysis treatment.

All statistical analyses were performed using the computing environment R (R Development Core Team, 2005) setting the significance level to 0.05.

## 3. Results

Between November 2010 and March 2011, a total of 92 (35.8%) patients out of the 257 who were receiving renal replacement treatment were identified as having a diagnosis of DM. Demographic and clinical characteristics of the sample are shown in Table 1. The mean age was 70.9 years (SD = 12.1 years), and the percentage of males was 62%, and all were of Caucasian origin. Eighty-one patients had type 2 DM (88%) and the mean diabetes duration was 22.3 years (SD = 12.2 years). The vast majority of patients were on haemodialysis (92.4%), and the mean duration of dialysis treatment was 4.8 years (SD = 4.3 years).

Concerning cardiovascular risk factors (Table 1), we observed that 43.5% of patients were current or former smokers, 68.5% of them had dyslipidaemia, and 84.8% of them had hypertension. In relation to late diabetic complications, DR was present in 62%; out of the 71 patients who underwent retinopathy exploration, 12% of them had amaurosis; and 9.8% and 22.8% had stroke and coronary heart disease, respectively.

The carotid ultrasound and PAD examinations were conducted in 81 patients. The mean IMT value was 0.90 cm (±0.17), and all patients had some grade of atheromatous disease, with 92.5% of them having nonstenotic carotid plaques. The prevalence of foot complications (Table 1) was, from the highest to the lowest, PN (89.1%), moderate or severe PAD (64.2%), foot deformities (54.3%), previous ulcer (19.6%), DF (17.4%), and amputations (16.3%) (Table 1). Finally, based on the IWGDF classification, out of 83 patients explored, 87% had some risk grade for suffering DF in the future.

From the bivariate analysis, the risk of ulceration was significantly increased in patients with DR (*p* = 0.0006) (Table 2) and was also increased in patients with stenosing carotid plaques (*p* = 0.05) and in patients with a previous stroke (*p* = 0.049). The risk of amputation was significantly increased in patients with DR (*p* = 0.02) and in patients with stenosing carotid plaques (*p* = 0.03) (Table 3). Furthermore, the multivariate analysis showed that foot ulceration was independently related to DR and stenosing carotid plaques (*p* = 0.004 and *p* = 0.023, resp.) and that DR was also an independent risk factor for lower-limb amputation (*p* = 0.013).

**Figure 1:**
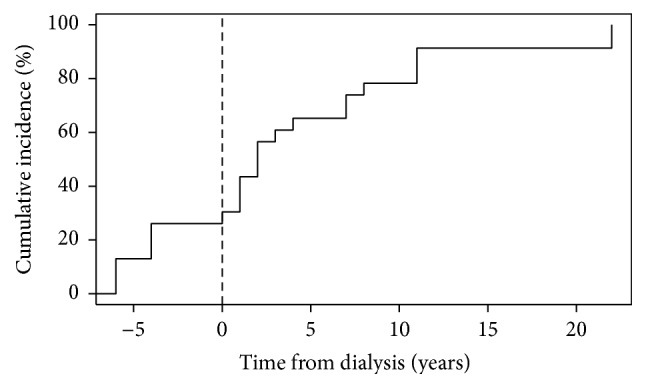
Cumulative incidence (%) of amputations depending on the time from initiating dialysis (years). All patients with an amputation were evaluated, including those who started dialysis after an amputation (with a negative number of years).

Finally, for those patients with an amputation, the relationship between the start of dialysis therapy and the cumulative incidence of amputations was assessed (Figure 1). The incidence increased progressively with time, showing an accumulated incidence of 18.7%, 37.5%, 43.8%, and 50% in the first, second, third, and fourth years after initiating dialysis, respectively.

## 4. Discussion

In this cross-sectional study in a representative population from our region, we observed that diabetic patients undergoing dialysis therapy had a high prevalence of DR, underlying PN, PAD, and foot deformities. Moreover, important vascular complications of diabetes, such as DR and stenosing carotid plaques, were shown to be independently associated with the risk of foot ulcers and lower-limb amputations.

The prevalence of PN in our sample was 89.1%, which is higher than the prevalence reported in some previous studies, ranging between 37% and 57% [8, 17, 18], but closer to other studies, with a prevalence between 77.2% and 80% [11, 19]. This higher prevalence could be due to the use of an ultrabiothesiometer, which is a precise and sensitive quantitative method, and differences between studies could be explained by disparities in the way the neurological assessment was performed and the instrument used, that is, loss of pressure sensation using a 10 g monofilament or vibration perception threshold with a neurothesiometer. However, even studies using the same instrument report wide differences: 3 of the studies using a monofilament reported a prevalence of 37% [18], 49% [17], and >77% [11] and 3 studies using a neurothesiometer reported a PN prevalence of 46% [18], 56.8% [8], and 80% [19]. Differences may be therefore due to unstandardised methodologies for assessment, for example, examiner experience, number and sites in the foot used, plantar or dorsal side of the foot, or definition of the pathological outcome.

The prevalence of PAD in our study (64.2%) is higher than the 45% reported by Jones et al. [18] and lower than the 73.5% reported by Kaminski et al. [17]. In the case of Jones et al., the study only explored 21 patients, and only 11 of them were diabetic; and in the Kaminski et al. study, vascular insufficiency was determined by manual palpation of both pedal pulses, and not by Doppler ultrasound.

The prevalence of foot deformities in our study (54.3%) was much lower than the prevalence described in two previous studies, which found a prevalence between 79% [18] and 71.4% [17], but higher than the 22% reported in another study [8]. The high association of foot lesions with advanced diabetic nephropathy may be explained by the long duration of diabetes, which predisposes to both nephropathy and foot lesions, and by the particularly high risk for nephropathic patients to develop macroangiopathic or neuropathic complications or a combination of both.

The frequency of current foot ulcer in our study was 17.4%, which is higher than the frequency reported in some studies (4%–12%) [17–19] and close to the figure described in another study comparing dialysis versus non-dialysis-treated patients (21% versus 4%) [8]. Moreover, the percentage of patients who had at least one episode of ulceration (i.e., current or previous) was similar to the one reported by Jones et al. (32%) [18]. Finally, the rate of amputations in this study (16.2%) was also in line with the 18% reported in another study [19] and again reported to be much higher than that in diabetic patients without dialysis (6.4%) [8].

According to the IWGDF risk scale, 87% of our patients had one or more risk factors for developing DF, which is very similar to the 89.8% described by Kaminski et al. [17], who found that the figure was lower in control groups (80% in DM patients and 71.8% in DM patients with end-stage renal disease). Our result is also close to the 93% and 96% risks reported by another group [8, 19].

The results of the multivariate analysis showed that the risk of foot ulceration was independently associated with DR and the presence of stenosing carotid plaques and that amputations were also independently associated with DR. The prevalence of DR among type 2 DM patients in haemodialysis has been previously reported to be between 70% and 79% [8, 20], which is only slightly higher than the 63% observed in our study. This is in line with the results of a retrospective study where the incidence of retinopathy was found to be significantly higher among subjects in haemodialysis who had a foot ulcer than that among those who did not (88% versus 54%) [21] and also with a recent meta-analysis that identified retinopathy as a risk factor for foot ulceration in adults with ESRD treated with dialysis [22]. Moreover, an association between DR and increased IMT as an early atherosclerosis marker in diabetic patients has been reported in two cross-sectional studies [15, 23]. Actually, diabetes is one of the most frequent and the most important clinical situations where endothelial dysfunction occurs [24]. The fact that we did not identify PN as a risk factor for ulceration and amputation could be explained by its high prevalence in our population (89.1%), acting as a confounding variable because PN is also correlated with the progression of chronic kidney disease and secondary to uraemia [6]. Conversely, DR is not confounded by uraemia and may be more specific and better reflect generalised microangiopathy than PN or diabetic nephropathy.

Finally, the pattern of cumulative incidence of amputations after the initiation of dialysis therapy in our cohort is similar to the one described by Game et al. [9], with an increasing incidence as treatment is prolonged in time. This could be explained by the combination of a progression of micro- and macrovascular complications while on dialysis and risk factors related to the progression of chronic kidney disease (e.g., malnutrition and chronic inflammation) and to dialysis therapy itself, which is an independent risk factor for major foot amputations irrespective of the presence of DM [25].

In our study, we did not find a relationship between foot ulceration and PAD. Although two previous studies have identified PAD as an independent risk factor for foot ulceration in patients with chronic kidney disease [26, 27], another study conducted in diabetic patients could not demonstrate this association [28]. However, none of these studies assessed differences among patients in dialysis. Indeed, our results are in agreement with another study reporting that PAD was not an independent risk factor after adjustment for dialysis therapy [8], although the same group had previously reported that it was independently associated in a multiracial sample [19]. The authors considered that the sample population and the inclusion of interrelated PAD and dialysis variables could explain the discrepancy of results between the two studies.

Foot deformities have been shown to increase plantar peak pressure and are considered as a factor for developing foot ulcers [14, 29]. However, and in line with a previous study reporting that foot deformities were less prevalent among patients in dialysis compared with no dialysis treatment [8], we could not find a direct relationship between foot deformities and foot ulceration or lower-limb amputations. Some of the most frequent reasons for the appearance of ulcers are frictions and repeated shoe-induced microtrauma, which can be largely prevented by the use of appropriate therapeutic footwear. In our catchment area, this type of footwear is reimbursed for patients who have one or more risk factors for ulceration, and it is often examined and prescribed at diabetic foot units. It is then possible that patients with foot deformities were already wearing adequate protective footwear, thus decreasing the chance of finding a relationship with foot ulcers and/or amputations.

The present study has strengths and limitations that must be acknowledged. The main strength of the study is that it is, as far as we are aware, the first one assessing the relationship between DF and atherosclerotic disease using the presence of atheromatous plaques by carotid artery ultrasound in diabetic patients receiving dialysis. Additionally, this is the first study published so far from a Spanish population. One limitation of the study is the relatively small sample size (92 patients), although it is representative of the area, encompassing the entire dialysis population of Lleida (Spain). Moreover, almost all patients were receiving haemodialysis, which prevented comparison with patients managed with peritoneal dialysis (*n* = 7) or even a separate analysis for each group. In addition, during PAD assessment, we did not quantitatively evaluate stenosis because the equipment we used did not record ultrasound waveforms, and we cannot discard that ABI values were in the normal range in patients with both arterial stiffness and stenosis, thus potentially underestimating the prevalence of PAD in our sample. Finally, not all patients were explored for all studied outcomes, which could have led in some cases to an underestimation of the true prevalence.

## 5. Conclusions

In our region, diabetic patients in dialysis treatment had a high prevalence of DF, and most of them had one or more risk factors for developing an ulcer in the future. DR and atheromatous disease were directly related to the appearance of ulcers and amputations.

Although this was not an interventional study, we believe that our findings have direct implications on foot care delivery, since there is a temporal association between the initiation of dialysis and the incidence of amputations. We suggest that all such patients should be considered at high risk and that appropriate foot care including preventive measures (e.g., scheduled foot examinations and education) and therapeutic intervention should be a must for all diabetic subjects in or initiating dialysis.

## Figures and Tables

**Table 1:** Demographic and clinical characteristics of patients included in the study.

Variable	Patients (*N* = 92)
Age (years), mean (SD)	70.9 (12.1)
Male sex, *n* (%)	57 (62)
Type 2 diabetes, *n* (%)	81 (88)
Diabetes duration (years), mean (SD)	22.3 (12.2)
Haemodialysis	85 (92.4)
Duration of dialysis (years), mean (SD)	4.8 (4.3)
Cardiovascular risk factors and long-term diabetes complications, *n* (%)	
Smoking status (current and former)	40 (43.5)
Dyslipidaemia	63 (68.5)
Hypertension	78 (84.8)
Retinopathy^*∗*^	57 (62.0)
Amaurosis^*∗*^	11 (12.0)
Stroke	9 (9.8)
Coronary heart disease	21 (22.8)
Foot complications, *n* (%)	
Peripheral neuropathy^†^	82 (89.1)
Peripheral artery disease^‡^	52 (64.2)
Foot deformities	50 (54.3)
Previous ulcer	18 (19.6)
Current ulcer: diabetic foot	16 (17.4)
Patients with amputations	15 (16.3)
Risk for diabetic foot (IWGDF), *n* (%)	
Risk 0	3 (3.3)
Risk 1	18 (19.6)
Risk 2	32 (34.8)
Risk 3	30 (32.6)
Not classified	7 (7.6)

^*∗*^Data available for 71 patients; ^†^data available for 90 patients; ^‡^data available for 81 patients.

SD, standard deviation.

**Table 2:** Bivariate and multivariate analysis for risk factors associated with foot ulceration.

Risk factor	Foot ulceration		Bivariate analysis		Multivariate analysis
	Yes	No	RR/mean diff. (95% CI)	*p* value	Adjusted *p* value
Gender, *n* (%)					
Male	21 (36.8)	37 (63.2)	1.84 (0.87–3.87)	0.09	0.60
Female	7 (20.0)	28 (80.0)			
Age (years), mean (SD)	70.1 (12.6)	71.3 (11.9)	−1.15 (−6.81–4.49)	0.75	
Diabetes, *n* (%)					
Type 1	4 (36.4)	7 (63.6)	1.22 (0.52–2.87)	0.65
Type 2	24 (29.6)	57 (70.4)		
Diabetes duration (years), mean (SD)	23.4 (11.0)	21.8 (12.8)	1.66 (−3.58–6.90)	0.43	
Dialysis duration, mean (SD)	4.9 (4.7)	4.7 (4.1)	0.15 (−1.91–2.22)	0.93	
Current or former smoker, *n* (%)					
Yes	14 (35)	26 (65.0)	1.3 (0.70–2.40)	0.41
No	14 (27.0)	38 (73.1)		
Dyslipidaemia, *n* (%)					
Yes	19 (30.2)	44 (69.8)	0.97 (0.50–1.88)	0.92
No	9 (31.0)	38 (73.1)		
Hypertension, *n* (%)					
Yes	25 (32.1)	53 (68.0)	1.49 (0.52–4.29)	0.45
No	3 (21.4)	11 (78.6)		
Retinopathy, *n* (%)					
Yes	26 (45.6)	31 (54.4)	6.84 (ne)	0.0006	0.004
No	0 (0.0)	14 (100)			
Amaurosis, *n* (%)					
Yes	3 (27.3)	8 (72.7)	0.72 (0.26–2.00)	0.54
No	23 (37.7)	38 (62.3)		
Stroke, *n* (%)					
Yes	6 (60.0)	4 (40.0)	2.23 (1.20–4.15)	0.04	0.73
No	22 (26.8)	38 (62.3)			
IHD, *n* (%)					
Yes	10 (47.6)	11 (52.4)	1.87 (1.03–3.42)	0.06	0.93
No	18 (25.4)	53 (74.6)			
PN					
Yes	25 (30.5)	57 (69.5)	2.43 (0.37–15.70)	0.32
No	1 (12.5)	7 (87.5)		
PAD, *n* (%)					
Yes	15 (29.4)	36 (70.6)	1.41 (0.58–3.43)	0.45
No	5 (20.8)	19 (79.2)		
Foot deformities, *n* (%)					
Yes	14 (28.0)	36 (72.0)	0.84 (0.45–1.55)	0.58
No	14 (33.3)	38 (66.7)		
AD, *n* (%)					
AD3	12 (34.3)	23 (65.7)	1.43 (0.71–2.85)	0.31
AD1 or AD2	11 (23.9)	35 (76.1)		
Carotid plaques, *n* (%)					
Stenosing	4 (66.7)	2 (33.3)	1.43 (0.71–2.85)	0.05	0.013
Nonstenosing	18 (23.9)	35 (76.1)			
IMT, cm, mean (SD)	0.9 (0.2)	0.9 (0.2)	0.05 (−0.05–0.16)	0.4	

AD, atheromatous disease; IHD, ischaemic heart disease; IMT, intima-media thickness; ne, nonestimable; PAD, peripheral artery disease; PN, peripheral neuropathy; RR, risk ratio.

**Table 3:** Bivariate and multivariate analysis for risk factors associated with lower-limb amputations.

Risk factor	Amputations		Bivariate analysis		Multivariate analysis
	Yes	No	RR/mean diff. (95% CI)	*p* value	Adjusted *p* value
Gender, *n* (%)					
Male	10 (17.5)	47 (82.5)	1.22 (0.45–3.29)	0.70
Female	5 (14.3)	30 (85.7)		
Age (years), mean (SD)	72.3 (10.3)	70.6 (12.4)	1.69 (−4.50–7.89)	0.74	
Diabetes, *n* (%)					
Type 1	1.0 (9.0)	10 (90.9)	0.52 (0.07–3.61)	0.78
Type 2	14 (17.3)	67 (82.7)		
Diabetes duration (years), mean (SD)	23.9 (13.6)	22.0 (12.0)	1.90 (−6.00–9.81)	0.79	
Dialysis duration, mean (SD)	5.5 (5.9)	4.6 (3.9)	0.81 (−2.55–4.18)	0.92	
Current or former smoker, *n* (%)					
Yes	6 (15)	34 (85)	0.86 (0.33–2.23)	0.78
No	9 (17.3)	43 (82.7)		
Dyslipidaemia, *n* (%)					
Yes	10 (15.9)	53 (84.1)	0.92 (0.34–2.45)	0.85
No	5 (17.2)	43 (82.8)		
Hypertension, *n* (%)					
Yes	14 (18.0)	64 (82.1)	2.51 (0.35–17.61)	0.35
No	1 (7.14)	13 (92.9)		
Retinopathy, *n* (%)					
Yes	15 (26.3)	42 (73.7)	3.94 (ne)	0.02	0.023
No	0 (0.0)	14 (100.0)			
Amaurosis, *n* (%)					
Yes	2 (18.8)	9 (81.8)	0.85 (0.22–3.26)	0.86
No	13 (12.3)	48 (78.7)		
Stroke, *n* (%)					
Yes	2 (20.0)	8 (80.0)	1.26 (0.33–4.79)	0.71
No	13 (15.9)	69 (84.1)		
IHD, *n* (%)					
Yes	4 (19.1)	17 (81.0)	1.21 (0.43–3.46)	0.69
No	11 (15.5)	60 (84.5)		
PN					
Yes	15 (8.3)	67 (81.7)	1.64 (ne)	0.21
No	0 (0.0)	8 (100.0)		
PAD, *n* (%)					
Yes	9 (17.7)	42 (83.4)	1.47 (0.34–6.29)	0.31
No	2 (8.3)	22 (91.7)		
Foot deformities, *n* (%)					
Yes	6 (12.0)	44 (88.0)	0.51	0.24
No	9 (21.4)	33 (78.6)		
AD, *n* (%)					
AD3	7 (20.0)	28 (80.0)	1.84 (0.63–5.31)	0.27
AD1 or AD2	5 (10.9)	41 (89.1)		
Carotid plaques, *n* (%)					
Stenosing	3 (50.0)	3 (50)	4.62 (1.64–13)	0.03	0.12
Nonstenosing	8 (10.8)	66 (89.2)			
IMT, mean (SD)	0.9 (0.2)	0.9 (0.2)	0.009 (−0.11–0.13)	0.97	

AD, atheromatous disease; IHD, ischaemic heart disease; IMT, intima-media thickness; ne, nonestimable; PAD, peripheral artery disease; PN, peripheral neuropathy; RR, risk ratio.
